# Maintenance metabolism and carbon fluxes in *Bacillus* species

**DOI:** 10.1186/1475-2859-7-19

**Published:** 2008-06-18

**Authors:** Simon Tännler, Seraina Decasper, Uwe Sauer

**Affiliations:** 1 Institute of Molecular Systems Biology, ETH Zurich, CH-8093 Zurich, Switzerland

## Abstract

**Background:**

Selection of an appropriate host organism is crucial for the economic success of biotechnological processes. A generally important selection criterion is a low maintenance energy metabolism to reduce non-productive consumption of substrate. We here investigated, whether various bacilli that are closely related to *Bacillus subtilis *are potential riboflavin production hosts with low maintenance metabolism.

**Results:**

While *B. subtilis *exhibited indeed the highest maintenance energy coefficient, *B. licheniformis *and *B. amyloliquefaciens *exhibited only statistically insignificantly reduced maintenance metabolism. Both *B. pumilus *and *B. subtilis *(natto) exhibited irregular growth patterns under glucose limitation such that the maintenance metabolism could not be determined. The sole exception with significantly reduced maintenance energy requirements was the *B. licheniformis *strain T380B. The frequently used *spo0A *mutation significantly increased the maintenance metabolism of *B. subtilis*.

At the level of ^13^C-detected intracellular fluxes, all investigated bacilli exhibited a significant flux through the pentose phosphate pathway, a prerequisite for efficient riboflavin production. Different from all other species, *B. subtilis *featured high respiratory tricarboxylic acid cycle fluxes in batch and chemostat cultures. In particular under glucose-limited conditions, this led to significant excess formation of NADPH of *B. subtilis*, while anabolic consumption was rather balanced with catabolic NADPH formation in the other bacilli.

**Conclusion:**

Despite its successful commercial production of riboflavin, *B. subtilis *does not seem to be the optimal cell factory from a bioenergetic point of view. The best choice of the investigated strains is the sporulation-deficient *B. licheniformis *T380B strain. Beside a low maintenance energy coefficient, this strain grows robustly under different conditions and exhibits only moderate acetate overflow, hence making it a promising production host for biochemicals and riboflavin in particular.

## Background

Optimal choice of the host organism for cell factory engineering is pivotal to the economic success of biotechnological processes. Typical host selection criteria are available genetic tools and methods, safety status, genome annotation, well-characterized process characteristics and scale-up behavior. For many products, in particular for bulk chemicals, cellular energetics is another key criterion whose impact becomes most relevant in near optimized processes [1]. In particular in the frequently employed fed-batch processes, where the production phase is prolonged by controlled feeding of the growth-limiting substrate, cells are placed in the domain of slow growth [2]. During this phase, cells devote increasing percentages of the consumed energy substrate not only for growth and product formation, but also for maintaining cellular homeostasis [3]. The relevant physiological parameter is the maintenance energy coefficient that specifies the amount of energy cells required for maintaining homeostasis in the absence of growth [4]. For optimal design of fed-batch processes with extended slow-growth conditions, choice of cell factories with appropriate cellular energetics and low maintenance energy coefficient thus becomes increasingly relevant [1].

An important class of industrial production hosts are the gram-positive bacilli as efficient secretors of enzymes and producers of antibiotics, purine nucleotides or vitamins. The industrially and scientifically most relevant representative of this group is *B. subtilis*, which is used extensively for enzyme and biochemicals production [5], in particular for the large-scale production of vitamin B_2_, riboflavin [6-8]. While *B. subtilis *fulfills most of the industrially desirable host criteria, it does not have a particularly low maintenance energy coefficient (0.44 mmol g^-1 ^h^-1 ^[9]) when compared to, for example, *B. licheniformis *(0.24 mmol g^-1 ^h^-1 ^[10]) or *Klebsiella aerogenes *(0.3 mmol g^-1 ^h^-1 ^[11]). Another industrially undesired feature of *B. subtilis *is its ability to form spores upon nutrient limitation [12]. To prevent sporulation during production, typically the first regulatory sporulation gene, *spo0A*, is deleted. Beyond sporulation, however, the Spo0A protein regulates several other cellular processes, and the *spo0A *mutation has been implied to increase the maintenance energy coefficient of riboflavin producing *B. subtilis *[9]. Here, we investigate specifically whether the *spo0A *mutation causes indeed increased maintenance demands.

Another important host characteristic is the distribution of intracellular fluxes as the starting point for metabolic engineering. For the production of riboflavin – synthesized from three pentose units, glycerin and two C1 units – significant fluxes through the oxidative pentose phosphate (PP) pathway deem intuitively advantageous [13]. While significant catabolic fluxes through the PP pathway are found in common lab strains of *Escherichia coli*, *B. subtilis *[14], *B. clausii *[15] and *B. megaterium *[16], many other bacteria exhibit exceptionally low PP pathway fluxes that only match the anabolic demand [14,17]. Partly related to the PP pathway flux, catabolic NADPH overproduction is another preferred host property because 3 NADPH are required per riboflavin molecule, and catabolic overproduction could be utilized for riboflavin biosynthesis [13]. To assess the potential of close *B. subtilis *relatives as alternative riboflavin cell factories, we quantified their distribution of fluxes as well as NADPH and maintenance metabolism. Specifically we focus on the growth rate-dependent flux distribution in glucose-limited chemostat cultures because of their similarity to the conditions during riboflavin production in fed-batch with continuously decreasing growth rates.

## Methods

### Bioreactors

A recently described mini-scale chemostat setup was used for parallel operation of 12 chemostats [18]. Briefly, bacteria were grown in sealed 17 ml Hungate tubes. To avoid stepwise feeding by dripping medium, the feed needle was placed directly in the culture liquid. The culture volume was kept constant at 10 ml by level control with a second needle placed at the desired level to suck off excessive culture broth. The medium removal pump was set to a speed of two culture volumes per minute, thereby also removing air from the headspace. The resulting underpressure caused air influx through a third needle, which was placed at the bottom of the reactor. The rising bubbles both aerated and mixed the cultures.

Due to sedimentation in the mini-scale reactors, *B. amyloliquefaciens *was the only species that was cultivated in 500 ml bioreactors (Infors AG, Switzerland). The cultivation volume of 250 ml was kept constant by level control. Throughout the cultivation, active mixing was achieved with a magnetic stirrer, and aeration was kept constant at two volume of air per volume of culture and minute.

### Species and growth conditions

Species used in this study are listed in Table 1. Frozen glycerol cultures were used to inoculate 5 ml Luria-Bertani (containing per liter: 5 g yeast extract, 10 g NaCl, 10 g tryptone) precultures, supplemented with antibiotics where necessary. From these LB precultures, we inoculated (1 to 500 diluted) 5 ml M9 minimal medium with 5 g/l glucose. From these overnight precultures, aerated Hungate tubes were inoculated at a ratio of 1 to 10. First, batch growth was allowed to proceed for 4 to 8 hours, and cultures were analyzed after continuous dilution of the medium for at least 6 volume changes at 37°C. For each steady state, a new chemostat culture was started from frozen stocks.

**Table 1:** Bacterial species used during this study

**Species/Mutant**	**Description**	**Genotype**	**Source**
*B. subtilis *wild type 168	cured of trp auxotrophy	trp+	DSM Nutritional Products Inc.
*B. subtilis spo0A*		trp+ spo0A-	DSM Nutritional Products Inc.
*B. subtilis sigE*		trp+ sigE-	DSM Nutritional Products Inc.
*B. licheniformis *T218a			DSM Nutritional Products Inc.
*B. licheniformis *T380B			DSM Nutritional Products Inc.
*B. subtilis natto *(DSM No. 4451)	cured of plasmids	bio- ade-	DSM Nutritional Products Inc.
*B. pumilus *(DSM No. 27)		bio- ade-	DSMZ^a^
*B. amyloliquefaciens*			DSM Nutritional Products Inc.

^a ^Deutsche Sammlung von Mikroorganismen und Zellkulturen GmbH

The M9 minimal medium was composed of the following components (per liter final volume): 5.64 g Na_2_HPO_4_, 3 g KH_2_PO_4_, 0.5 g NaCl, 1 g NH_4_Cl, 0.246 g MgSO_4_·7H_2_O, 0.014 g CaCl_2_, at pH 7.4 and 10 ml trace element solution containing (per liter) 1.35 g FeCl_2_6H_2_O, 0.1 g MnCl_2_·H_2_O, 0.17 g ZnCl_2_, 0.043 g CuCl_2_·2H_2_O, 0.06 g CoCl_2_·6H_2_O, 0.06 g Na_2_MoO_4_·2H_2_O [19]. Filter-sterilized glucose was added to a final concentration of 1 g per liter. For ^13^C-labeling experiments, glucose was added either as a mixture of 20% (wt/wt) U-^13^C-labeled isotope isomer (99%; Cambridge Isotope Laboratories, Andover, MA) and 80% (wt/wt) natural glucose or as 100% 1-^13^C-labeled isotope isomer (99%; Cambridge, Isotope Laboratories, Andover, MA). Labeled glucose was used throughout the experiment, including the batch phase.

Batch experiments were done at 250 rpm in 500 ml baffled shake flask with a culture volume of 50 ml and 5 g/L glucose for non-labeled experiments and 30 ml volume and 3 g/L glucose for labeling experiments. The medium composition and preculturing were identical to the chemostat experiments. For *B. subtilis *natto and *B. pumilus*, biotin and adenine were added from sterile stock solutions to final concentrations of 0.1 μg/ml and 20 μg/ml, respectively.

### Analytical procedures and physiological parameters

Cell growth was monitored by determining optical density at 600 nm (OD_600_). Glucose and acetate concentrations in culture supernatants were determined by using refractive index (RI) and UV detectors, respectively, on a HPLC system (Agilent/Hewlett Packard Series 1100) with an Aminex HPX-87H column (Biorad, Hercules, CA).

For continuous cultures, all physiological parameters were determined during steady state between 5 to 7 volume changes after inoculation. Since dilution and thus growth rate are constant in chemostat cultures, consumption and production rates were determined from the differences between substrate (S) and product (P) concentrations in the feed medium and culture supernatant. The relationship q_S(or P) _= ΔS (or P) (D/X) we calculated specific production and consumption rates, where X is the biomass concentration. For determination of maintenance energy coefficients glucose consumption rates (q_glc_) were plotted against dilution rates. The maintenance energy coefficients were determined as the y-axis intercept of the weighted least square (WLS) regression line using the SPSS statistical software package (SPSS Inc. Chicago, IL).

In batch culture, the growth rate (μ) was determined as the coefficient of the log-linear regression of OD_600 _versus time. The biomass yield on the substrate (Y_X/S_) was determined as the coefficient of a linear regression of biomass concentration versus substrate concentration during the exponential phase. The specific substrate consumption rate was determined as the coefficient of a linear regression of substrate concentrations versus biomass concentrations multiplied by μ. The same relationship holds for the specific rate of formation of (by-)products.

To calculate specific biomass yields, maintenance coefficients, consumption and production rates, a correlation factor for cellular dry weight (CDW) to OD_600 _was used. To determine the cellular dry weight, 10 ml culture broth was transferred into preweighted 15 ml glass tubes and centrifuged for 10 min at 3000 g at 4°C. The pellets were washed twice with 0.9% NaCl and dried at 105°C for 24 h to constant weight. Except for the *spo0A *mutant, a single correlation factor (gCDW/OD) was used for all dilution rates (D) of a given species: *i.e.*, *B. subtilis *wild type 0.48, *B. subtilis sigE *0.41, *B. licheniformis *T218a 0.55, *B. licheniformis *T380B 0.48, *B. subtilis *natto 0.54, *B. pumilus *0.61 and *B. amyloliquefaciens *0.36. For the *B. subtilis spo0A *mutant the CDW-to-OD correlation varied with the growth rate (data not shown), hence we determined the correlation factors for this mutant at dilution rates of 0.05, 0.1, 0.2 and 0.4 h^-1 ^from mini-scale cultivations. These values were confirmed in a 1 l working volume stirred tank reactor (data not shown). The resulting correlation factors for the *spo0A *mutant were 0.39 gCDW/OD for D of 0.05 h^-1^, 0.51 gCDW/OD for D of 0.2 to 0.3 h^-1 ^and 0.44 gCDW/OD for D of 0.4 to 0.5 h^-1^.

### Metabolic flux ratio analysis

Samples for gas chromatography-mass spectrometry (GC-MS) analysis were prepared as described previously [20,21]. Briefly, biomass from ^13^C-labeled chemostat cultures was harvested after stable OD_600 _for at least 2 volume changes (at least 6 volume changes in total). For shake flask experiments, cells were harvested during mid-exponential growth at an OD_600 _of 1–1.5. Cell pellets were hydrolyzed in 6 M HCl at 105°C in sealed microtubes overnight. Hydrolyzates were then dried under a constant air stream at 60°C. Derivatization was carried out at 85°C in 30 μl dimethylformamide (Fluka, Switzerland) and 30 μl *N*-(*tert*-butyldimethylsilyl)-*N*-methyl-trifluoroacetamide with 1% (v/v) *tert*-butyldimethylchlorosilane (Fluka, Switzerland) for 60 min. Derivatized amino acids were analyzed on a series 8000 GC, combined with an MD 800 mass spectrometer (Fisons Instruments, Beverly, MA). The GC-MS-derived mass isotope distributions were then analyzed using the software FiatFlux [22]. Briefly, from the mass distributions of the amino acids the ^13^C-labeling patterns of their related precursor molecules in central metabolism were inferred. A set of probabilistic equations and the mass distributions of selected amino acid fragments were then combined to calculate the relative contribution of converging fluxes to a given metabolite pool [21].

### ^13^C-constrained metabolic flux analysis

Intracellular fluxes were estimated by fitting a flux distribution to the above flux ratios and quantitative physiological data within a stoichiometric model described by [23] using the software FiatFlux [22]. Reaction reversibilities were chosen according to [23]. The reaction matrix contained 24 unknown fluxes and 21 metabolite balances, including balances for glucose, acetate, CO_2_, O_2_, and the cofactors NADH and NADPH. Precursor requirements for biomass formation were taken from [23]. To solve this under-determined system of linear equations, five additional constraints in the form of the above calculated flux ratios were used, i.e. serine derived through glycolysis, oxaloacetate originating from pyruvate, phosphoenolpyruvate (PEP) originating from oxaloacetate, upper and lower bound of pyruvate originating from malate and PEP derived through the PP pathway. The sum of the weighed square residuals of the constraints form both metabolite balances and flux ratios was minimized using the MATLAB (The Mathworks) function fmincon and the residuals were weighed by dividing through the experimental error [24]. The computation was repeated at least five times with randomly chosen initial flux distributions to ensure identification of the global minimum.

## Results

### Physiology and fluxes during batch growth on glucose

A desirable physiological characteristic of cell factories is rapid and fully respiratory growth. For riboflavin production, in particular, an active PP pathway is expected to be a second relevant criterion because the product is primarily synthesized from pentose units. To investigate the potential suitability of *B. subtilis *168 and four closely related bacilli as cell factories for biochemicals production, we determined their physiological parameters and fluxes during exponential growth in glucose batch cultures. The subspecies *B. subtilis *natto is phylogenetically the closest relative to *B. subtilis*, followed by *B. amyloliquefaciens *and *B. licheniformis*, while *B. pumilus *is somewhat more distal [25].

**Figure 1:**
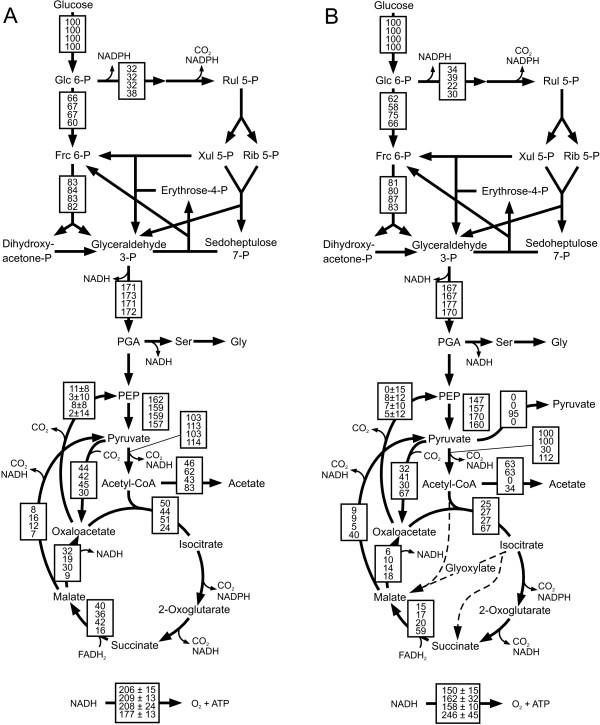
**Relative distribution of absolute fluxes in glucose batch cultures of A) *B. subtilis *wild type (top), *spo0A *(middle-top), *sigE *(middle-bottom) mutants and natto (bottom) and B) *B. licheniformis *T218a (top), *B. licheniformis *T380B (middle-top), *B. amyloliquefaciens *(middle-bottom) and *B. pumilus *(bottom)**. Fluxes were determined by ^13^C-constrained flux analysis from two separate experiments with 100% [1-^13^C]glucose and with a mixture of 20% [U-^13^C] and 80% unlabeled glucose (Additional file 2) along with the physiological data given in Table 2. Generally, the 95% confidence intervals were between 10 and 15% of the values shown for the major fluxes. Larger confidence intervals were estimated for reactions with low fluxes. To facilitate cross species comparison, fluxes are normalized to the glucose consumption rates given in Table 2. Arrowheads indicate flux direction.

The ^13^C-flux data obtained for *B. subtilis *168 compare favorably with previously reported data from other *B. subtilis *strains [26] and also with reported metabolic flux ratios for the type strain 168 [27]. To extend flux analysis to other bacilli, we first verified their network topologies by comparison with the KEGG database [28]. All four genomes contained the genes for the reactions of the *B. subtilis *network, with the exception of an absent 6-phosphogluconate dehydrogenase in *B. pumilus *(Additional file 1). Since our labeling experiments with both [U-^13^C] and [1-^13^C] glucose demonstrated significant PP pathway fluxes also in *B. pumilus *(Figure 1), we assumed the presence of a 6-phosphogluconate dehydrogenase.

None of the investigated bacilli contain genes encoding enzymes of the Entner-Doudoroff pathway and absence of this pathway was also confirmed from the obtained ^13^C-labeling patterns (data not shown). In addition to the *B. subtilis *network, the *B. licheniformis *genome contains the two glyoxylate shunt-encoding genes, which enables *B. licheniformis *to grow on two-carbon units such as acetate or 2,3-butanediol [29]. For the experiments shown here, however, we verified the absence of in vivo glyoxylate shunt fluxes from the calculated fraction of labeled CO_2 _[18]. This result is consistent with the normally observed glucose repression of this shunt [30].

During unlimited growth on glucose, intracellular fluxes (Figure 1) and physiology (Table 2) varied significantly among the investigated bacilli. The growth physiology of *B. subtilis *natto was similar to *B. subtilis*, with the obvious difference of elevated acetate secretion and a concomitantly lower TCA cycle flux. *B. amyloliquefaciens *and *B. pumilus *both grew rather slowly and were the most distinct from the others. *B. amyloliquefaciens *has the lowest biomass yield and low relative flux through the PP pathway. Moreover, its overflow metabolism is entirely different, secreting massive amounts of pyruvate instead of acetate. *B. pumilus *exhibited mostly respiratory metabolism with high relative TCA cycle fluxes. As a peculiarity, it has unusually high fluxes through the pyruvate shunt from malate to pyruvate and on to oxaloacetate that are indirectly inferred from the ^13^C data.

**Table 2:** Physiological data of glucose minimal medium batch cultures

**Species**	**Growth rate**	**Yield**	**Glucose consumption rate**	**Acetate production rate**	**Pyruvate production rate**
	h^-1^	g_CDW_/g_glc_	mmol g^-1 ^h^-1^	mmol g^-1 ^h^-1^	mmol g^-1 ^h^-1^
*B. subtilis wild type*	0.67 ± 0.02^a^	0.44 ± 0.03^a^	8.71 ± 0.64^a^	4.28 ± 0.29^a^	n/a
*B. subtilis spo0A*	0.71 ± 0.03	0.33 ± 0.02	11.30 ± 0.14	7.02 ± 0.21	n/a
*B. subtilis sigE*	0.67 ± 0.01	0.48 ± 0.06	7.90 ± 0.74	3.60 ± 0.08	n/a
*B. amyloliquefaciens*	0.32 ± 0.01	0.23 ± 0.02	6.05 ± 0.24	n/a	5.45 ± 0.05
*B. licheniformis *T218a	0.37 ± 0.02	0.44 ± 0.01	4.63 ± 0.24	3.03 ± 0.20	0.22 ± 0.03
*B. licheniformis *T380B	0.49 ± 0.02	0.41 ± 0.03	6.67 ± 0.31	4.17 ± 0.94	0.40 ± 0.01
*B. pumilus*	0.28 ± 0.02	0.33 ± 0.04	4.17 ± 0.33	1.40 ± 0.60	n/a
*B. subtilis *natto	0.69 ± 0.02	0.35 ± 0.05	10.83 ± 0.82	9.60 ± 1.10	n/a

^a ^Value refer to standard deviation from at least two independent experiments. n/a – not applicable

The two *B. licheniformis *strains are derivatives of the parent strain T5, a producer of thermostable alpha-amylase [31,32]. Specifically, strain T380B is a sporulation-deficient, direct descendant of T218a that was obtained after multiple rounds of classical mutagenesis and screening for traits that improved alpha-amylase production. Under the investigated conditions, however, less than 10 μg/ml protein was secreted in the medium, which did not significantly affect the mass balances. Both strains grow at intermediate specific growth rates with relatively high biomass yields (Table 2). In terms of intracellular fluxes, both *B. licheniformis *strains have about half of the TCA cycle flux of *B. subtilis*.

In addition to *B. subtilis *wild type 168, we analyzed also the otherwise isogenic *B. subtilis *168 mutants *spo0A *and *sigE*. In particular, we were interested whether the frequently used *spo0A *mutation has unfavorable effects on metabolism through one of its many pleiotrophic effects rather than through the intended block of sporulation. As control for block of sporulation we used the *sigE *mutant that cannot enter stage III of sporulation [12]. The phenotypic differences of *B. subtilis *wild type and *spo0A *compared to earlier work [27] is mainly due to different cultivation systems that lead to higher growth rates in the present experiments and possibly also to differences in strain background. While the *sigE *mutation is phenotypically silent under this condition, the *spo0A *mutant has a significantly increased glucose consumption and acetate formation rate (Table 2). The consequence is a strongly induced overflow metabolism and hence a reduced yield of biomass (Table 2), indicating *spo0A *as a suboptimal choice to suppress sporulation.

### Influence of growth rate on fluxes

**Figure 2:**
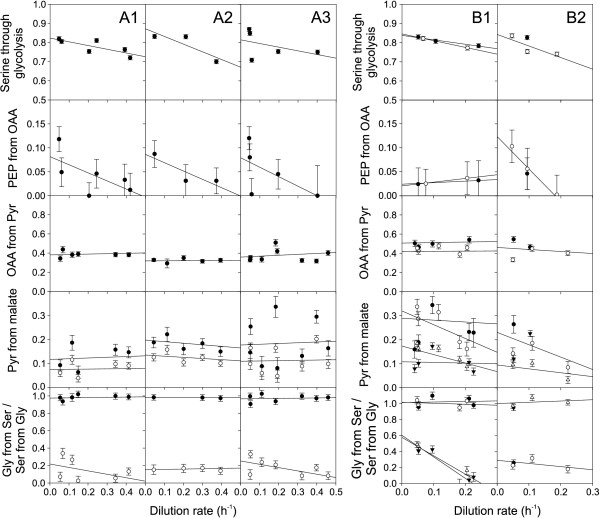
**Metabolic flux ratios in glucose-limited chemostat cultures at different dilution rates of A1) *B. subtilis *wild type, A2) spo0A and A3) sigE mutant, B1) *B. licheniformis *T218a (filled symbols) and T380B (open symbols) and B2) *B. subtilis *natto (open symbols) and *B. pumilus *(filled symbols)**. The fraction of serine derived through glycolysis and the fraction of PEP originating from oxaloacetate were obtained from 100% [1-^13^C]glucose. All other ratios were obtained from experiments with 20% [U-^13^C]glucose and 80% natural glucose. The experimental error was estimated from redundant mass distribution [20].

Since most industrial processes are based on carbon source-limited fed-batches at low growth rates, we quantified growth rate dependent physiology and fluxes in 10-ml glucose-limited chemostat cultures [18]. In contrast to the discontinuously decreasing TCA cycle flux with decreasing growth rate that was described for glucose-limited *E. coli *chemostat cultures [18], all ratios of intracellular fluxes in *B. subtilis *and its sporulation mutants changed continuously or remained constant with decreasing growth rates (Figure 2). While the anaplerotic flux ratio of oxaloacetate derived from pyruvate remained almost invariant, the proportion of glycolytic versus PP pathway (serine derived through glycolysis) and the gluconeogenic flux through the PEP carboxykinase (PEP originating from oxaloacetate) increased continuously with decreasing growth rate (Figure 2). The latter result is consistent with the notion of decreasing catabolite repression at the severe glucose limitation at low dilution rates [33].

**Figure 3:**
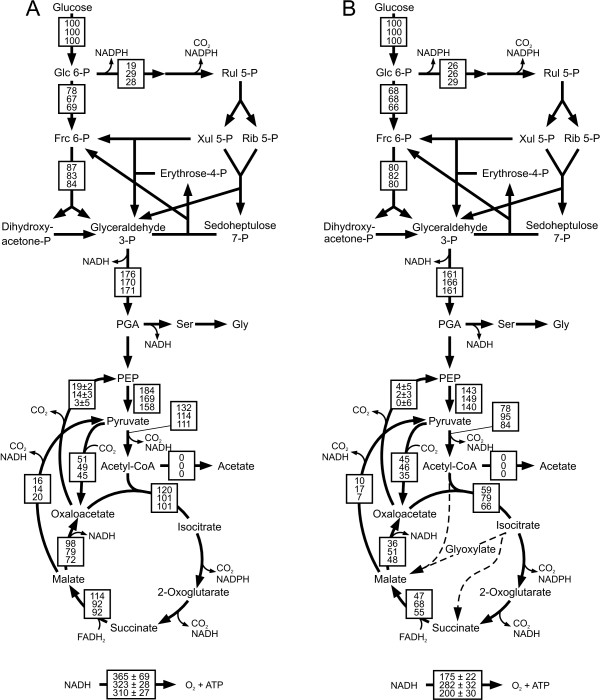
**Relative distribution of absolute fluxes of A) *B. subtilis *wild type at dilution rates of 0.05 h^-1 ^(top), 0.2 h^-1 ^(middle) and 0.4 h^-1 ^(bottom) and B) *B. licheniformis *T218a (top), *B. licheniformis *T380B (middle) and *B. subtilis *natto (bottom) at dilution rate 0.2 h^-1^**. Fluxes were determined by ^13^C-constrained flux analysis from two separate experiments with 100% [1-^13^C]glucose and with a mixture of 20% [U-^13^C] and 80% unlabeled glucose (Additional file 2) along with the physiological data of the chemostat experiments (additional files 5 and 7). Generally, the 95% confidence intervals were between 10 and 15% of the values shown for the major fluxes. Larger confidence intervals were estimated for reactions with low fluxes. To facilitate cross species comparison, fluxes are normalized to the glucose consumption rates given in (additional files 5 and 7). Arrowheads indicate flux directions.

Absolute in vivo fluxes were then quantified by integrating the determined extracellular fluxes (Additional file 4) and the intracellular flux ratios (Additional file 2) by ^13^C-constrained flux analysis [24]. In contrast to the flux distribution during unrestricted growth on glucose (Figure 1A) glucose-limited chemostat cultures of *B. subtilis *show i) no overflow metabolism, ii) about doubled relative TCA cycle flux that remained stable over different dilution rates, and iii) significant gluconeogenic flux through the PEP carboxykinase at the lowest dilution rate (Figure 3A).

The other bacilli exhibited generally rather similar trends in their flux ratios as seen for *B. subtilis *(Figure 2A and 2B). The sole exception was the in vivo PEP carboxykinase activity (PEP from oxaloacetate) of *B. licheniformis*. While this flux ratio was high at low dilution rates but absent at higher values in all other investigated bacilli, we observed a significant but constant fraction of PEP molecules originating from oxaloacetate in both *B. licheniformis *strains. At the representative dilution rate of 0.2 h^-1^, we then calculated flux distributions for both *B. licheniformis *strains and *B. subtilis *natto (Figure 3B). The main differences to *B. subtilis *were the rather low respiratory TCA cycle fluxes in all three species.

### NADPH metabolism

**Figure 4:**
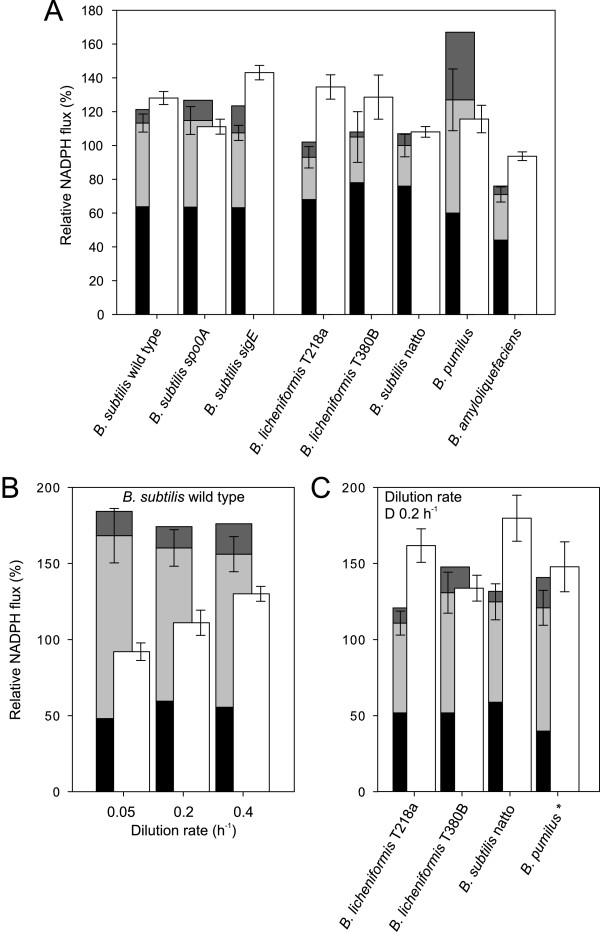
**Comparison of NADPH production and consumption during unrestricted (A) and glucose-limited (B and C) growth**. Black, light gray and dark gray bars represent the estimated relative NADPH formation by the PP pathway, isocitrate dehydrogenase and malic enzyme, respectively. NADPH formation via malic enzyme is an upper bound, since all investigated species possess at least two malic enzymes with different cofactor specificities. Results from *B. pumilus *marked with an asterisk refer to a dilution rate of 0.1 h^-1^. Error bars represent the summed confidence of the flux estimates of the PP pathway and the TCA cycle. The white bars represent the NADPH consumption rate of biomass with errors representing the standard deviation of the experimentally determined growth rate.

Since the distribution of fluxes differed significantly in the investigated species, we were interested whether their catabolic NADPH formation matched the anabolic demand. For this purpose, we quantified NADPH formation from the previously quantified carbon fluxes through the NADPH-dependent reactions catalyzed by the oxidative PP pathway and the isocitrate dehydrogenase. The anabolic demand of NADPH was directly quantified from the known biochemical requirements of NADPH for growth-dependent macromolecules biosynthesis [34]. Within the resolution of the analysis most *B. subtilis *strains exhibited balanced NADPH production and consumption during unrestricted growth on glucose (Figure 4A). Under glucose limitation however, there was clearly a catabolic overproduction for *B. subtilis *wild type (Figure 4B) and the two mutants (data not shown), as was reported earlier [35].

The NADPH balance was significantly different in the other bacilli. Firstly, the summed catabolic NADPH formation of both *B. licheniformis *strains was clearly insufficient to match the anabolic demand in batch and in one case also in chemostat culture (Figure 4A and 4C). This consistent catabolic underproduction was primarily caused by the rather low TCA cycle fluxes in *B. licheniformis*. Secondly, no species appeared to exhibit a catabolic NADPH overproduction like *B. subtilis *under any of the investigated conditions (data partially shown in Figure 4A and 4C). The sole exception might be the batch-grown *B. pumilus *due to its exceptionally high malic enzyme flux (Figure 4A). Since it is unclear which of the two malic enzymes with different cofactor specificities was active, however, this result is not conclusive.

### Maintenance metabolism

**Figure 5:**
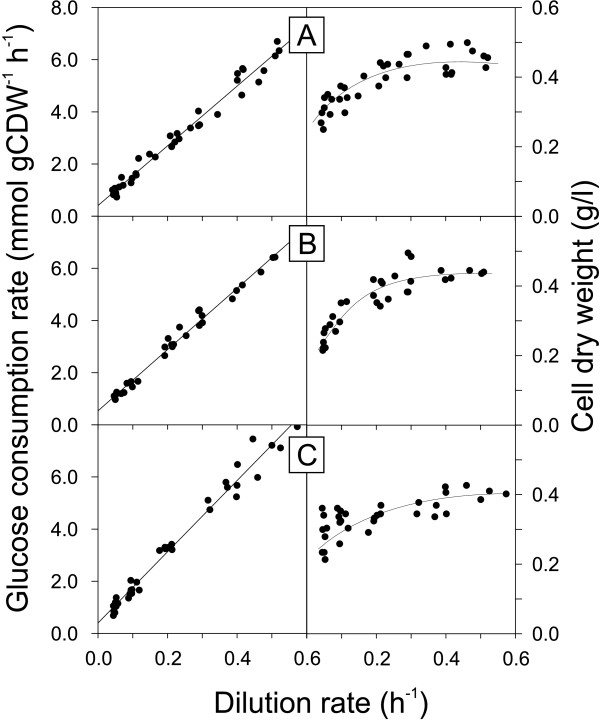
**Specific glucose consumption rate and biomass concentration as a function of dilution rate in glucose-limited chemostat cultures of *B. subtilis *wild type 168 (A), and its *spo0A *(B) and *sigE *(C) mutants**. Trend lines in the left column represent the weighed least square regression. Trend lines in the right column were drawn by hand.

**Figure 6:**
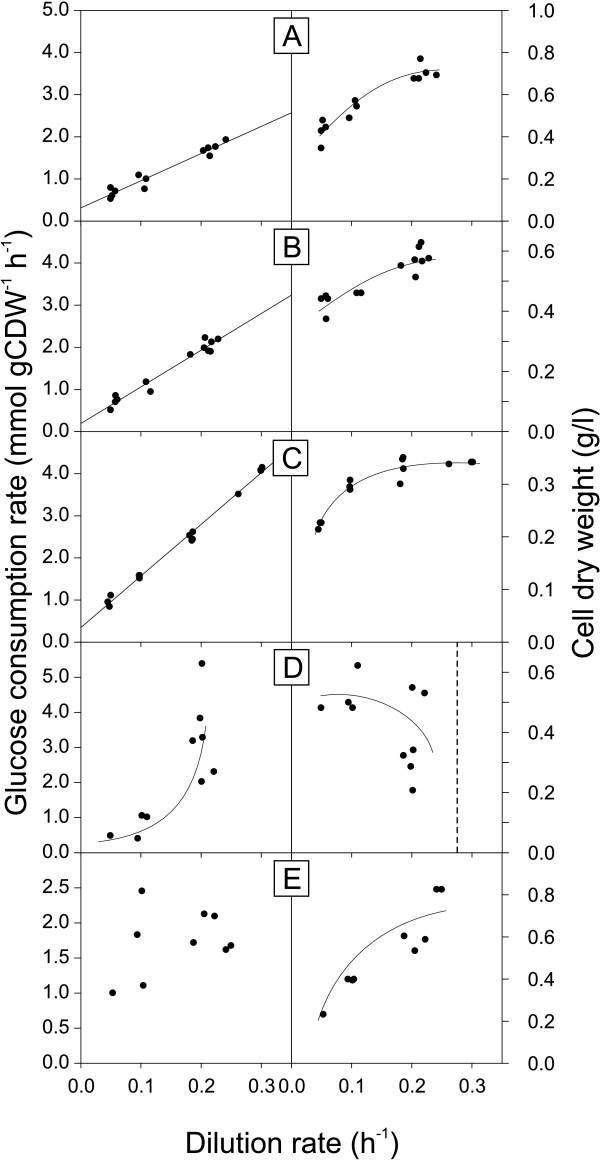
**Specific glucose consumption rate and biomass concentration as a function of dilution rate in glucose-limited chemostat cultures of *B. licheniformis *T218a (A), *B. licheniformis *T380B (B), *B. amyloliquefaciens *(C), *B. pumilus *(D) and *B. subtilis *natto (E)**. Trend lines of A, B and C of the left column represent a weighed least square regression, all other trend lines were drawn by hand. Due to sedimentation in the 10 ml chemostats, *B. amyloliquefaciens *(C) was cultivated in actively stirred and aerated reactor with 250 ml culture volume.

Finally, to assess the bioenergetic suitability of the different species as biochemicals production host, we determined their maintenance metabolism; i.e. the amount of energy required to maintain cellular homeostasis in the absence of growth. Typically this non-growth-associated maintenance energy coefficient is quantified by applying Pirt's chemostat model [4]:

$$q_{glc}=\frac{\mu }{Y_{glc}^{\max}}+m_{glc}$$

where *q*_*glc *_is the specific glucose consumption rate, *m*_*glc *_the maintenance energy coefficient and $Y_{glc}^{\max}$ the maximum molar growth yield. Using the physiological data from the above described chemostat cultures, we found, consistent with the Pirt model, a linear dependency of the glucose consumption rate over the whole range of tested dilution rates for *B. subtilis *(Figure 5), *B. licheniformis *and *B. amyloliquefaciens *(Figure 6). For the *B. subtilis spo0A *mutant we determined growth rate specific OD_600 _to cell dry weight conversion factors, because they did not remain constant, in contrast to the other species.

For these bacilli with linear dependencies of the specific glucose consumption rate with dilution rate, the maintenance energy coefficients were determined as the intercept of the weighted least square regression line with the y-axis (Table 3). The determined coefficient of 0.39 mmol g(cdw)^-1 ^h^-1 ^for *B. subtilis *wild type 168 compares favorably to the previously reported 0.44 mmol g(cdw)^-1 ^h^-1 ^of the related *B. subtilis *wild type 1012 [9]. The *spo0A *mutant, but not the *sigE *mutant, exhibited an increased maintenance coefficient of 0.49 mmol g(cdw)^-1 ^h^-1^, which is qualitatively consistent with the previously reported high maintenance energy coefficient of riboflavin-producing *spo0A *mutants of *B. subtilis *[9,36]. Together, these results provide strong evidence that the *spo0A *mutation has a stimulating and biotechnologically undesirable effect on maintenance metabolism.

**Table 3:** Maintenance energy coefficients in different bacilli.

**Species**	**Maintenance coefficient**^a^	
	mmol g^-1 ^h^-1^	R^2^
*B. subtilis *wild type	0.39 ± 0.08^b^	0.978^c^
*B. subtilis spo0A*	0.49 ± 0.06	0.976
*B. subtilis sigE*	0.33 ± 0.09	0.979
*B. amyloliquefaciens*	0.35 ± 0.13	0.993
*B. licheniformis *T218a	0.30 ± 0.18	0.949
*B. licheniformis *T380B	0.20 ± 0.17	0.958

^a^values were calculated by weighted least square (WLS) regression from data shown in Figure 5 and Figure 6; ^b^95% confidence interval of a weight least square (WLS) linear regression analysis; ^c^R^2 ^represents the square of the correlation coefficient of the linear regression.

Both *B. licheniformis *strains and *B. amyloliquefaciens *exhibited lower maintenance energy coefficients than *B. subtilis *wild type (Table 3). In most cases, however, the difference was minor and not statistically significant. The sole exception with significantly reduced maintenance energy requirements was *B. licheniformis *T380B, despite a large confidence interval. For *B. pumilus *and *B. subtilis *natto, the maintenance coefficients could not be determined because their specific glucose consumption rates did not show a linear relationship with dilution rate (Figure 6). The reasons are strong fluctuations in their steady state biomass concentrations, which, at least for *B. pumilus*, are related to dilution rates close to the maximum specific growth rate (Table 2).

## Discussion

The requirements for successful biotechnological production hosts are diverse and depend greatly on the product of interest. Here we characterized several important metabolic properties that are critical for biochemicals production in different bacilli: i) general growth physiology, ii) maintenance metabolism, and iii) a favorable distribution of intracellular fluxes for production of the vitamin riboflavin, including catabolic overproduction of NADPH.

From a bioenergetic point of view, *B. subtilis *is not a preferred cell factory for riboflavin production because it had the highest maintenance energy requirements of all bacilli investigated here. The maintenance advantage of most other bacilli, however, was rather small and was counterbalanced by several other disadvantages such as slow growth and/or metabolism that render them even less favorable to production than *B. subtilis*. The by far best choice would be *B. licheniformis *T380B, which had only about half of the maintenance energy coefficient of *B. subtilis *wild type. Since this strain grows also robustly in different cultivation devices and shows only moderate acetate overflow, it is a promising cell factory host for biochemicals production in general, and riboflavin production in particular.

Just how important are these differences in maintenance metabolism on a process scale? In fermentation for penicillin production, for example, up to 70% of the carbon source is utilized for maintenance [37]. Similarly during industrial riboflavin production with the production strain RB50::pRF69, maintenance metabolism expends on the order of 45% of the consumed glucose (based on published data from [36] with a maintenance coefficient of 0.67 g g(cdw)^-1 ^h^-1^). Reduction of the maintenance coefficient by 50% through a host switch, for example to *B. licheniformis *T380B, seems possible as demonstrated here. Provided a similar riboflavin formation is engineered, this could reduce the glucose consumption in the overall riboflavin fed-batch process by 15–20%. Even at otherwise unaltered riboflavin titers, this constitutes a major yield improvement with high commercial relevance for a low-cost, feed chemical like riboflavin because substrate costs are the key driver in this process.

Obviously, changing production hosts is a major undertaking that must be justified by the potential gains. Even within one strain line, however, maintenance metabolism can become a key process factor. Firstly, maintenance metabolism is not a constant but can be affected by various genetic manipulations. While a similar maintenance energy coefficient as reported here was described in a different *B. subtilis *wild-type (0.44 mmol g(cdw)^-1 ^h^-1^), its riboflavin producing counterpart exhibited a 50% higher coefficient of 0.66 mmol g(cdw)^-1 ^h^-1 ^[9]. This is even more surprising when considering that this particular strain was only subtly engineered; *i.e. *it contained only a single copy of the modified *rib *operon, the deregulating *ribC *mutation, and the *spo0A *mutation. A key finding in the present work is that the frequently used *spo0A *mutation [6] does not only influence the host physiology under all conditions tested, but that it is also a major contributor to increased maintenance metabolism. The riboflavin manipulations themselves do not appear to be of particular relevance because the industrial RB50 strain with its much higher production level and multiple copies of the *rib *operon in the genome as well as further uncharacterized mutations had the virtually identical maintenance coefficient of 0.67 mmol g(cdw)^-1 ^h^-1 ^as the above subtly engineered strain [9,36]. Further down the sporulation regulation cascade, the *sigE *mutation, in contrast, also prevents sporulation but had no detectable physiological phenotype and a wild-type-like maintenance coefficient. It thus appears to be the preferred choice for *B. subtilis*, and this seemingly simple change in sporulation mutations has the potential to significantly decrease glucose consumption in the above mentioned riboflavin fed-batch process [36] because the *spo0A *mutation alone increased the maintenance coefficient of the wild-type by 20%.

The intracellular fluxes reported here for *B. subtilis *are consistent with previous reports on particular growth conditions in disparate studies [9,13,26,27,34,35,38-40]. In particular, we observed absence of overflow metabolism and high relative TCA cycle fluxes in glucose-limited chemostats. In consequence, these fluxes lead to a significant excess formation of NADPH in chemostats. Compared to the other investigated bacilli, *B. subtilis *exhibited rather high respiratory TCA cycle fluxes under all conditions. At a dilution rate of D 0.2 h^-1^, the absolute TCA flux of *B. subtilis *was 2.9 ± 0.45 mmol g^-1 ^h^-1 ^while the maximum values of other bacilli were in the range from 1.3 ± 0.26 to 1.9 ± 0.29 mmol g^-1 ^h^-1 ^and thus significantly lower. As a result of their comparatively low TCA cycle fluxes, the other bacilli produced much less NADPH in the isocitrate dehydrogenase reaction. Consequently catabolic NADPH formation was either balanced or even insufficient to match the anabolic demand in all non-*B. subtilis *species. Although not a rigorous criterion like maintenance metabolism, the tendency towards catabolic overproduction of NADPH renders *B. subtilis *more attractive for riboflavin production than the other species.

To balance NADPH metabolism, *B. subtilis *thus requires a transhydrogenase-like biochemical mechanism to counteract catabolic NADPH overproduction, at least in chemostat culture. Most of the other bacilli, in contrast, require the opposite mechanism, i.e. an additional supply of NADPH. How this may be achieved remains elusive at this point because these species contain no homologue of the NADPH-producing transhydrogenase PntAB of *E. coli *[41].

## Authors' contributions

SD carried out most of the experimental work, ST participated in experimental work, carried out the data analysis and drafted the manuscript, US designed and coordinated the study and contributed to the writing of the manuscript. All authors read and approved the final manuscript.

## Supplementary Material

Additional file 1 Network comparison of *B. subtilis*, *B. licheniformis*, *B. amyloliquefaciens *and *B. pumilus *based on the KEGG database Click here for file

Additional file 2 flux ratios of glucose batch experiments Click here for file

Additional file 3 flux ratios from chemostat experiments Click here for file

Additional file 4 Absolute net and relative fluxes of *B. subtilis *wild type, *spo0A *and *sigE *mutants during batch cultivation Click here for file

Additional file 5 Absolute net and relative fluxes of *B. subtilis *at different dilution rates. Click here for file

Additional file 6 Absolute net and relative fluxes of different bacilli during batch cultivation Click here for file

Additional file 7 Absolute net and relative fluxes of different bacilli at dilution rate D 0.2 h^-1^ Click here for file
